# Beyond αβ T cells: NK, iNKT, and γδT cell biology in leukemic patients and potential for off-the-shelf adoptive cell therapies for AML

**DOI:** 10.3389/fimmu.2023.1202950

**Published:** 2023-08-15

**Authors:** Andrew Kent, Lyndsey S. Crump, Eduardo Davila

**Affiliations:** ^1^Division of Medical Oncology, Department of Medicine, University of Colorado, Aurora, CO, United States; ^2^Human Immunology and Immunotherapy Initiative, University of Colorado, Aurora, CO, United States; ^3^Department of Medicine, University of Colorado Comprehensive Cancer Center, Aurora, CO, United States; ^4^TrAMPoline Pharma, Inc., Aurora, CO, United States; ^5^Department of Medicine, University of Colorado, Aurora, CO, United States

**Keywords:** NK cell, iNKT cell, gdT cell, adoptive cell therapy, acute myeloid leukemia, immune cell engineering

## Abstract

Acute myeloid leukemia (AML) remains an elusive disease to treat, let alone cure, even after highly intensive therapies such as stem cell transplants. Adoptive cell therapeutic strategies based on conventional alpha beta (αβ)T cells are an active area of research in myeloid neoplasms given their remarkable success in other hematologic malignancies, particularly B-cell-derived acute lymphoid leukemia, myeloma, and lymphomas. Several limitations have hindered clinical application of adoptive cell therapies in AML including lack of leukemia-specific antigens, on-target-off-leukemic toxicity, immunosuppressive microenvironments, and leukemic stem cell populations elusive to immune recognition and destruction. While there are promising T cell-based therapies including chimeric antigen receptor (CAR)-T designs under development, other cytotoxic lymphocyte cell subsets have unique phenotypes and capabilities that might be of additional benefit in AML treatment. Of particular interest are the natural killer (NK) and unconventional T cells known as invariant natural killer T (iNKT) and gamma delta (γδ) T cells. NK, iNKT, and γδT cells exhibit intrinsic anti-malignant properties, potential for alloreactivity, and human leukocyte-antigen (HLA)-independent function. Here we review the biology of each of these unconventional cytotoxic lymphocyte cell types and compare and contrast their strengths and limitations as the basis for adoptive cell therapies for AML.

## Introduction

Since their first successful implementation in the late 1960s, stem cell transplants (SCTs) were long regarded as the only means to cure acute myeloid leukemia (AML), particularly patients with high-risk cytogenetics (1). Although associated with lower treatment-related mortality, autologous transplants are associated with much higher rates of relapse than allogeneic transplants (2). Furthermore, a higher incidence of graft-versus-host disease (GVHD), a lymphocyte-mediated process, correlates with improved outcomes after SCTs (3). These and other observations led to the proposition that it was not the cytotoxic conditioning regimens used pre-transplantation that allowed for cure, but rather the unique ability of lymphocytes, particularly donor-derived lymphocytes, to recognize and destroy residual host-derived AML cells. With the accumulation of now decades of supporting evidence, particularly the characterization of leukemia-specific cytotoxicity of donor T cells, and identification of unique host minor histocompatibility antigens (miHA), a lymphocyte-dependent graft-versus-leukemic (GVL) effect is now widely accepted and mechanistically represents one of the earliest cell-based immunotherapies (4–6).

Unfortunately, subsequent attempts to further harness the GVL effect for AML treatment have had underwhelming results. Donor-lymphocyte infusions (DLIs), although capable of inducing durable remissions in up to 75% of relapsing post-transplant chronic myeloid leukemia (CML) patients, only result in remissions rates in the 15-24% range in relapsing post-transplant AML patients and are associated with increased incidence of GVHD (7). The high failure rate of lymphocyte infusions at the time of AML relapse suggests the original anti-leukemic effect of lymphocytes is lost as the myeloid leukemia progresses. There are several possible mechanisms for this immune escape including selection for immune-evasive leukemic clones, impaired cytotoxicity due to lymphocyte-intrinsic changes, development of an immunosuppressive tumor microenvironment (TME) in the bone marrow or other lymphoid organs, or some combination of all the above. Strategies to better recruit and activate immune cells and thereby destroy residual AML are an important area of research.

Due to their relative abundance in circulation and proven contribution to GVL effects through recognition of mismatched HLA and miHA through their T cell receptor (TCR), research focused on T cells expressing the alpha beta (αβ)T cell receptors and these cells have dominated the AML immunotherapy literature. However, αβT cells require stringent HLA-matching to function in a recipient, are major contributors to GVHD, and have a propensity for exhaustion and anergy with chronic stimulation. Because of these issues, alternate cytotoxic lymphocyte cell types may be better substrates for engineering and adoptive cell therapies in certain contexts. Natural killer (NK), invariant natural killer T (iNKT), and γδT cells have unique properties that may be beneficial for specific clinical applications. NK cells lack a TCR and are considered to be part of the innate immune system. iNKT cells exhibit properties of both innate and adaptive immunity, expressing both innate receptors and a semi-variant TCR. Gamma delta (γδ)T cells are cytotoxic with both major histocompatibility complex (MHC)-dependent and MHC-independent activity. Thus, in combination with αβ T cells, these three cell types represent the full spectrum from innate to adaptive T cell immunity. Currently, there are no assays that quantify the number of NK, iNKT, or γδT subsets in the cellular products, and no studies have rigorously assessed their contribution to the therapeutic success of DLI or chimeric antigen receptor (CAR)-T treatment. It is possible that any of these cell types play an integral part in tumor cell destruction in extant adoptive approaches including SCTs, DLIs, and CAR-T therapies despite representing a significant minority of total cells in each. In the case of AML, each of these cell types, especially NK cells, have been shown to have unique contributions to disease pathology at baseline and some promise in early pre-clinical and clinical studies.

**Figure 1:**
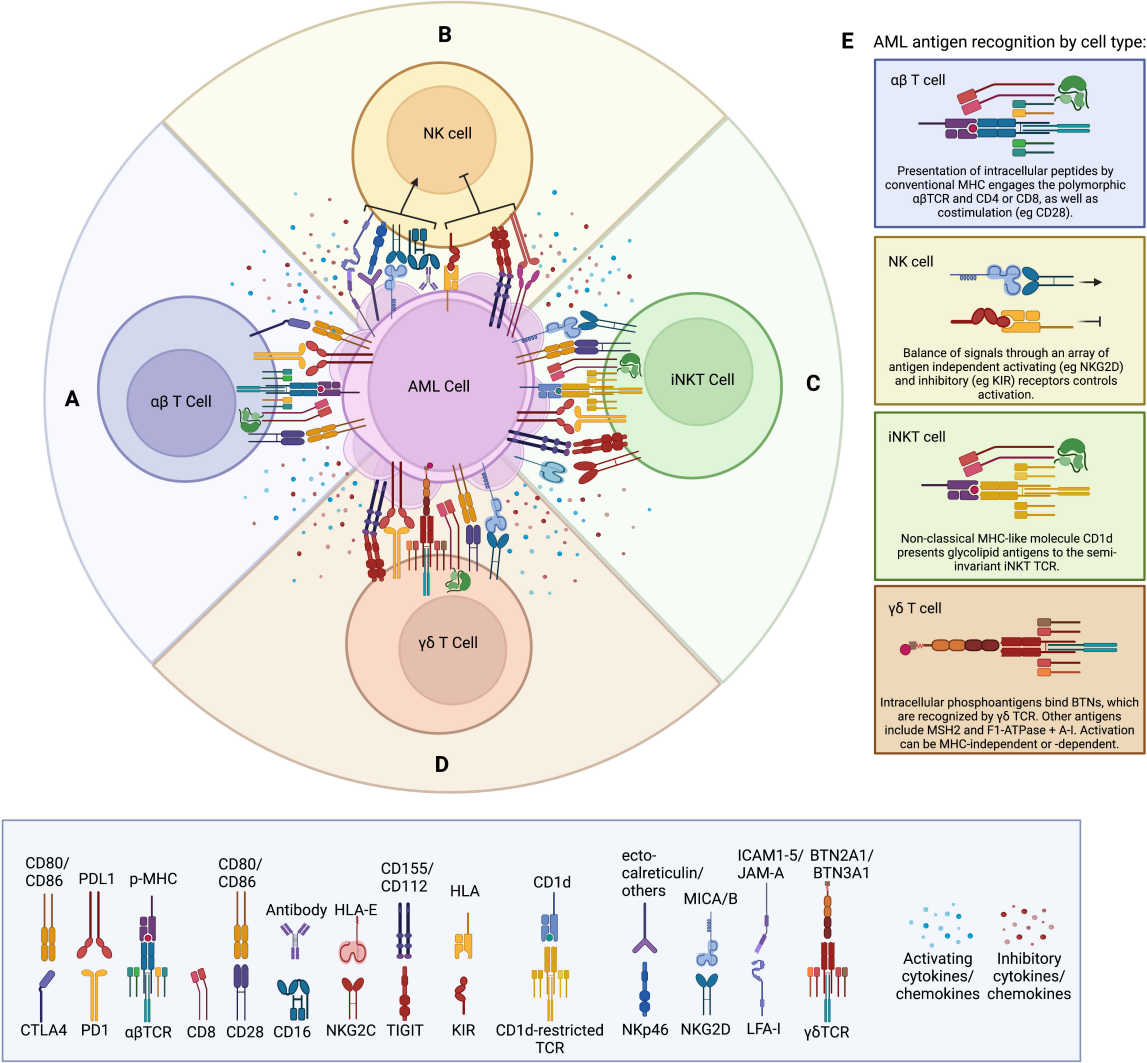
**(A)** Schematic of conventional αβTCR activating receptors including the αβTCR complex, CD8, and CD28, as well as inhibitory receptors such as CTLA4 and PD-1. Soluble factors produced within the tumor microenvironment (such as IL-6, IL-10, TGFβ, and VEGF) are thought to suppress cytotoxic T cell responses. Soluble factors produced by the T cell are dependent on subtype (e.g. granzyme B and perforin by CD8; IFNγ, by Th1; IL-4, IL-5, and IL-10 by Th2; IL-10 and TGFβ by Treg). **(B)** Schematic of NK regulation via a balance of non-antigen dependent activating and inhibitory receptors. Suppressive soluble factors produced by the tumor include IL-6, IL-10, TGFβ, and VEGF. Classic soluble factors produced by NK cells include IFNγ, perforin, granzyme B, and TNFα. **(C)** Schematic of iNKT cell activation through the non-polymorphic iNKT TCR binding glycolipid presented by MHC, as well as non-antigen dependent activating and inhibitory receptors common to NK cells. Soluble factors produced by iNKT cells include cytotoxic molecules [perforin, granzyme **(B)**], as well as cytokines dependent on subset. **(D)** Schematic of γδT cell depicting the unique γδTCR, which can recognize antigens in both an HLA-dependent and HLA-independent manner. γδT cells express the classic T cell marker CD28, as well as CD8 in a population of cells. The inhibitory receptors PD-1 and TIGIT can be upregulated on γδT cells during exhaustion. γδT cells can also express cytotoxicity-associated NK cell receptors such as NKG2D, and they produce an array of secreted factors, including perforin, granzyme B, IFNγ, and TNFα. **(E)** Details of unique antigen recognition machinery present on each cell type as indicated.

**Figure 2:**
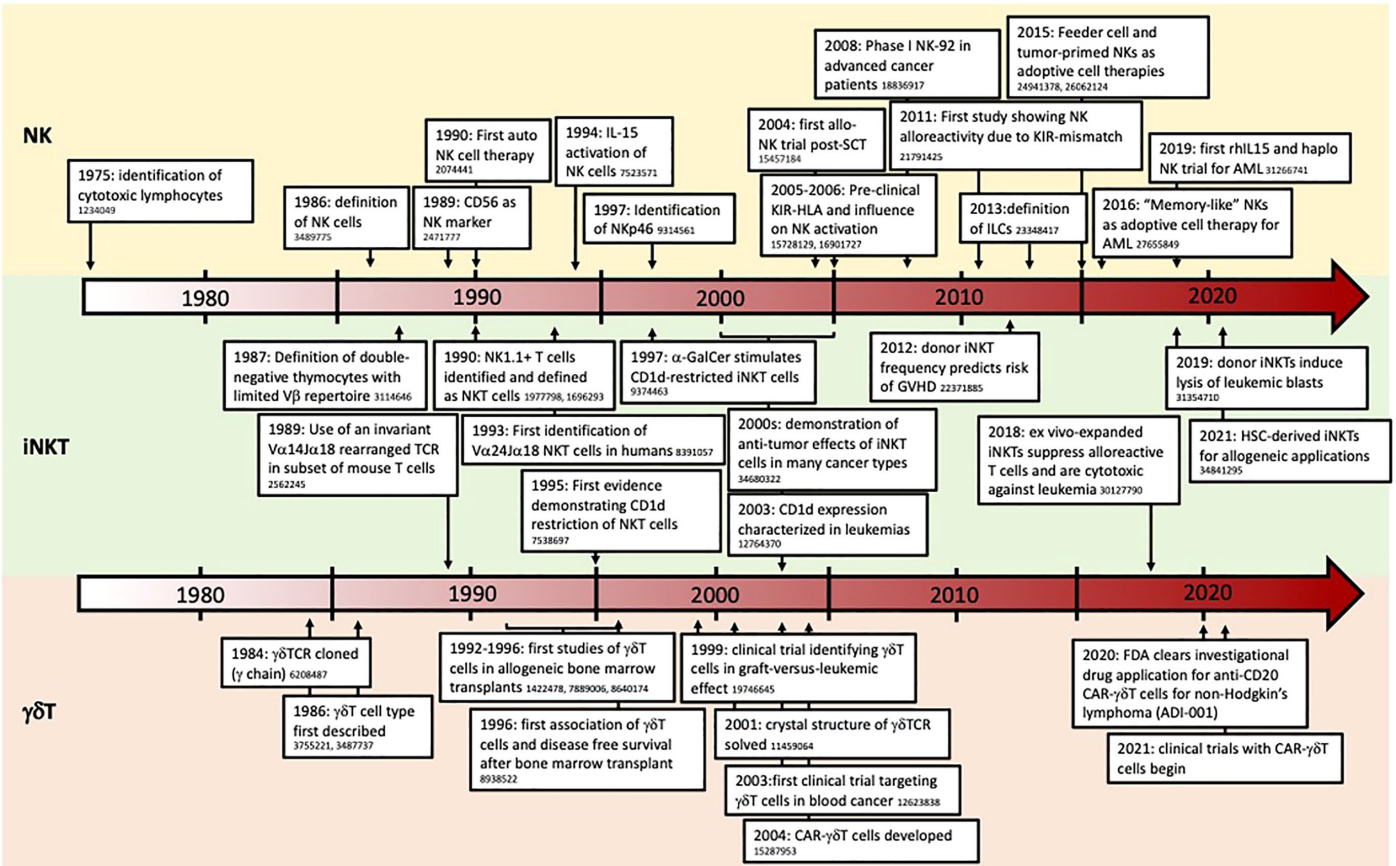
Timeline of major developments in NK, iNKT, and γδT cell biology, and applications in AML. Numbers indicate relevant pubmed IDs (PMIDs).

In this review, we compare the biology of αβT, NK, iNKT, and γδT cells, particularly the mechanisms by which they are activated or inhibited and how their phenotypes are altered in AML patients. We then summarize the published literature on approaches being studied to exploit each of these cell types for AML treatment in various clinical settings and strategies used to engineer them to augment their clinical efficacy. A summary of our cell type comparisons is represented schematically in **Figure 1**, in tabular format in **Table 1**, and described in detail in the following sections. **Figure 1E** describes the unique antigen-dependent or independent activation of each cell type. A timeline of important events in our understanding of NK, iNKT, and γδT cell biology, and their use as cell therapies in AML, is provided in **Figure 2**. All ongoing and completed phase II and III clinical trials using these cell types in AML are provided in **Table 2**.

**Table 1:** Comparison of major qualities of αβT, NK, iNKT, and γδT cells with regards to manufacturing and biology for applications in cell therapies.

Cell type:	αβ T cell	NK cell	iNKT cell	γδ cell
% of PBMCs	70%	5-20%	0.1-0.5%	1-10%
% in bone marrow	3-8%	10%	minimal	<5% of CD3+ cells
**Activating receptors:**	**αβ T cell**	**NK cell**	**iNKT cell**	**γδ cell**
TCR	Diverse	No	glycolipid/CD1d-directed	Diverse
TCR ligands	Highly diverse peptides presented by MHC	NA	alpha-GalCer, other endogenous and foreign glycolipids	Limited subset
HLA restriction	Yes. CD4: MHC-II, CD8: MHC-I	KIR specific to certain HLA alleles, but not dependent on HLA for activation	non-classical CD1d-restricted	Some, but largely independent of HLA activation
CD4/CD8	60%/40%	30% CD8+, no CD4+	Inhuman: CD4+ 50%/CD8+ 20%/DP 10%/DN 20%	<1%/30%
CD3	100%: required for TCR expression	20-30%, unclear function in absence of TCR	100%: required for TCR expression and signaling	100% with low and high expressing subsets
Costim	Yes: CD28, 41BB, etc	NA	Yes: CD28, 41BB, etc	Yes: CD28, CD27, etc
Other activating receptors	Require TCR	Diverse	TCR-dependent and TCR-independent activation potential	TCR-dependent and TCR-independent activation potential
**Inhibitory receptors:**	**αβ T cell**	**NK cell**	**iNKT cell**	**γδ cell**
CTLA4	+++	+	++	+ (rare)
KIR	-	++++	++	+
PD1	++++	++	++	++
LAG3	++	++	++	+
TIM3	++	++	++	++
TIGIT	++	+++	++	++
**Biology:**	**αβ T cell**	**NK cell**	**iNKT cell**	**γδ cell**
Expansion upon activation	Significant, but risk conversion to exhaustive/anergic phenotype	Modest	Minimal	Significant, but risk conversion to exhaustive/anergic phenotype
Persistence	Can be longterm with memory responses	Most short-lived - few weeks. Longer-lived memory-like NK cells can be generated after IL-12/15/18 exposure ex vivo	Short-lived responses, questionable memory-like phenotype in certain settings	Can be longterm with memory responses
Cytotoxicity	Potent, particularly CD8+ subset	Potent, rapid	Potent, rapid	Potent, rapid
Cytokine production	Diverse: Th1/Th2/Th17/Treg	Mostly IFN-y	Diverse: iNKT1, iNKT2, iNKT17 in mice, CD4+/CD8+/DN in humans	Diverse: IFN-γ/IL-10/TNF-α
Exhaustion/Anergy	Significant, well defined, after repeat antigen exposure	Poorly defined	Transient for ~2 months after antigen exposure	Exhaustion following persistent antigen stimulation
Relationship to GVHD after SCT	Cause GVHD	Mostly suppress GVHD, may exacerbate GVHD in specific settings	Suppress GVHD	Minimal risk of GVHD
**Engineering:**	**αβ T cell**	**NK cell**	**iNKT cell**	**γδ cell**
Isolation	CD3, CD4/CD8 selection from PBMCs	PBMC negative selection or CD56-positive selection	Selection of CD1d-alpha-GalCer tetramer positive cells. Can also be generated from CD34+ HSCs	γδ TCR selection from PBMCs
Expansion ex vivo	CD3/CD28 - bead or plate bound, with other cytokines for particular phenotypes	Thousands fold with mbIL-21 engineered feeder cells, commercial bead-based kits also availabe	alpha-GalCer stimulation using irradiated PBMC APCs and IL-2	Approaches using phosphoantigens or bisphosphonates are in development
Expansion in vivo	Lymphocyte depleting pre-conditioning, IL-2	Limited normally, increased with memory-like subsets	Limited	Treatment with zoledronic acid and IL-2
Persistence	Memory cells longterm	Limited normally, longer with IL-12/15/18 pre-exposure	Not well studied	Reports of up to 28 days but not well studied
Freeze/thaw	Feasible	Feasible	Feasible	Feasible with ~50% yield
Autologous application	Yes, endogenous TCR silencing or deletion with CARs, engineered TCRs	Yes	Yes	Possible but efforts largely focused on allogenic applications
Allogeneic application	Possible but requires HLA-matching, endogenous TCR deletion	Anti-leukemic activity improved with HLA-mismatching	Yes: Universal CD1d-restricted TCR	Trials in progress - HLA-matching encouraged, donor α/β TCR deletion

+ = low expression, ++ = moderate expression, +++ = high expression. NA, not applicable. Shading in table cells differentiate cell types.

**Table 2:** All ongoing and completed phase II and III clinical trials involving NK and gdT cells in AML. Of note, there are no current iNKT trials in AML. Terminated and withdrawn trials are excluded.

NCT Number	Study Title	Study Status	Brief Summary	Conditions	Sponsor	Phase	Start Date	Completion Date	Associated Results Publications
NCT04024761	A Phase 1 Trial of CIML NK Cell Infusion for Myeloid Disease Relapse After Hematopoietic Cell Transplantation	ACTIVE_NOT_ RECRUITING	Cytokine induced memory-like natural killer (CIML NK) cells combined with IL-2 in adult patients (18 years of age or older) with Acute Myeloid Leukemia (AML), Myelodysplastic Syndrome (MDS) and Myeloproliferative Neoplasms (MPN) who relapse after haploidentical hematopoietic cell transplantation (haplo-HCT) or HLA matched stem cells.	Myeloid leukemias	Dana-Farber Cancer Institute	I	August-19	December-24	
NCT03050216	QUILT-3.033: Haplo NK With SQ ALT-803 for Adults With Relapsed or Refractory AML	COMPLETED	This is a multi-institutional Simon’s optimal two-stage phase II trial of CD3/CD19 depleted, ALT-803 activated, haploidentical donor NK cells and subcutaneous ALT-803 given after lymphodepleting chemotherapy (CY/FLU) for the treatment of refractory or released acute myelogenous leukemia (AML).	AML	Masonic Cancer Center, University of Minnesota	II	May-17	December-19	PMID: 34797911
NCT02763475	NK Cells as Consolidation Therapy of Acute Myeloid Leukemia in Children/Adolescents	COMPLETED	Anti-relapse prophylactic activity of inoculating Natural Killer (NK) cells as consolidation therapy of acute myeloid leukemia in pediatric patients with cytologic remission. The patients included have intermediate risk of relapse and no indication for allogeneic hematopoietic stem cell transplantation. After the standard induction and consolidation chemotherapy treatment, patients will receive five days of fludarabine. Two different NK cells infusions will be performed within one week (day 0 and 7), with Interleukin 2 (IL-2) infusion.	AML	Instituto de Investigacion Hospital Universitario La Paz	II	May-16	August-20	PMID: 33610500
NCT00526292	Chemotherapy and a Donor Natural Killer Cell Infusion in Treating Patients With Relapsed or Persistent Leukemia or Myelodysplastic Syndrome After a Donor Stem Cell Transplant	COMPLETED	Chemotherapy followed by Haplo-NK cells refractory or at relapse after haplo-identical transplant.	MDS/AML	Memorial Sloan Kettering Cancer Center	II	August-07	July-15	PMID: 26772158
NCT02395822	MT2014-25: Haplo NK With SQ IL-15 in Adult Relapsed or Refractory AML Patients	COMPLETED	CD3/CD19 depleted, IL-15 activated, donor natural killer (NK) cells in adults and subcutaneous IL-15 given after a preparative regimen for the treatment of relapsed or refractory acute myelogenous leukemia (AML). The primary objective is to study the potential efficacy of NK cells and IL-15 to achieve complete remission while maintaining safety.	AML	Masonic Cancer Center, University of Minnesota	II	October-15	December-16	PMID: 31266741
NCT01370213	NK Cell Based Non-Myeloablative Transplantation in Acute Myeloid Diseases	COMPLETED	NK-cell based nonmyeloablative haploidentical transplantation for the treatment of high-risk acute myeloid diseases. Enrollment will use a two-stage design. Stage 1 will enroll 15 patients unless an early stopping rule is met. If 9 or more of these first 15 patients achieve leukemia free neutrophil engraftment at day +28 accrual will move to stage 2. In stage 2, an additional 28 patients will be enrolled for a total of 43 patients.	MDS/AML	Masonic Cancer Center, University of Minnesota	II	September-11	April-17	
NCT03349502	MG4101 for Refractory or Relapsed AML	COMPLETED	Investigate the efficacy and safety of allogeneic natural killer cell (MG4101). After lymphodepletion with fludarabine and cyclophosphamide, the patient will receive MG4101. Each cycle consists of 28 days, and a total of 2 cycles of MG4101 will be administered with IL-2 to activate the study drug. The efficacy of MG4101 will be evaluated after 8 weeks from the first day of treatment.	AML	Seoul National University Hospital	II	November-17	April-20	https://doi.org/10.1182/blood-2021-152137
NCT00703820	Clofarabine Plus Cytarabine Versus Conventional Induction Therapy And A Study Of NK Cell Transplantation In Newly Diagnosed Acute Myeloid Leukemia	COMPLETED	Assess the feasibility and efficacy of a novel form of therapy-haploidentical NK cell transplantation-in patients with standard-risk AML. In addition, we will investigate the efficacy of clofarabine + cytarabine (Clo/AraC) in newly diagnosed patients with AML and attempt to optimize outcome through the use of MRD-adapted therapy and further improvements in supportive care.	AML	St. Jude Children’s Research Hospital	III	August-08	August-20	PMID: 30894213
NCT01904136	Natural Killer Cells Before and After Donor Stem Cell Transplant in Treating Patients With Acute Myeloid Leukemia, Myelodysplastic Syndrome, or Chronic Myelogenous Leukemia	COMPLETED	Side effects and best dose of natural killer cells before and after donor stem cell transplant and to see how well they work in treating patients with acute myeloid leukemia, myelodysplastic syndrome, or chronic myelogenous leukemia. Giving chemotherapy with or without total body irradiation before a donor peripheral blood stem cell or bone marrow transplant helps stop the growth of cancer cells.	Myeloid malignancies	M.D. Anderson Cancer Center	I/II	April-14	February-22	PMID: 34312462
NCT04836390	Donor-Derived Ex-Vivo Expanded Natural Killer Cell Infusions in Children and Young Adults With High Risk Acute Myeloid Leukemia Receiving Myeloablative HLA-Haploidentical Hematopoietic Cell Transplant	ENROLLING_BY_ INVITATION	Three fixed-dose infusions of donor-derived NK cells in patients post-haploidentical stem cell transplant.	AML	Children’s Hospital Los Angeles	II	August-21	May-28	
NCT05319249	Natural Killer Cell Immunotherapy in Combination With PARP-inhibition in Acute Myeloid Leukemia	NOT_YET_ RECRUITING	Evaluate the combination of NK cell therapy and PARP inhibition by Talazoparib in patients with poor prognosis AML as characterized by Minimal Residual Disease (MRD) or overt relapse with less than 20% bone marrow blasts.	AML	German Cancer Research Center	I/II	June-23	June-27	
NCT05470140	A Phase 1 Study of WU-NK-101 in Patients With Relapsed or Refractory (R/R) Acute Myeloid Leukemia (AML)	NOT_YET_ RECRUITING	This study is a Phase 1, open-label, dose escalation, and cohort expansion study designed to characterize the safety, tolerability, pharmacokinetics, pharmacodynamics, immunogenicity, and preliminary anti-leukemic activity of WU-NK-101 in R/R AML.	AML	Wugen, Inc.	I	July-23	December-25	
NCT05834244	A Phase Ib Trial of Azacitidine, Venetoclax and Allogeneic NK Cells for Acute Myeloid Leukemia (ADVENT-AML)	NOT_YET_ RECRUITING	To learn if adding a healthy person’s natural killer (NK) cells to the combination of Azacitidine and Venetoclax can help to control AML. NK cells are cancer- and infection-fighting immune cells.	AML	M.D. Anderson Cancer Center	I	September-23	June-28	
NCT05215015	Study of Anti-CD33/CLL1 CAR-NK in Acute Myeloid Leukemia	RECRUITING	This is an open-label, nonrandomized, investigator-initiated clinical trial to evaluate the safety, tolerability, pharmacokinetics, and efficacy of anti-CD33/CLL1 CAR-NK cell injection in patients with acute myeloid leukemia (AML), and to determine PK parameters, maximum tolerated dose (MTD), and phase II recommended dose (RP2D) for subjects receiving CAR-NK cell injection.	AML	Wuxi People’s Hospital	I/II	November-20	November-22	
NCT05333705	Donor Immune Cell Therapy for Acute Myeloid Leukemia	RECRUITING	This study aims to introduce a new technology of donor NK cell infusion. NK cells defend against viruses and cancer cells *in vivo* whereas this effect declines in patients with tumors. In this study, NK cells will be separated from donated peripheral blood or umbilical cord blood. Eligible NK cells will be infused to patients with Acute myeloid leukemia (AML).	AML	First Affiliated Hospital Xi’an Jiaotong University	I	July-21	September-24	
NCT04632316	A Trial to Evaluate the Safety and Efficacy of oNKord¬Æ in Subjects With Acute Myeloid Leukemia	RECRUITING	WiNK is a Phase I/IIa trial to evaluate the safety and efficacy of oNKord¬Æ in 33 adults with acute myeloid leukemia (AML) who are in morphologic complete remission with residual measurable disease and who are currently not proceeding to allogeneic hematopoietic stem cell transplantation.	AML	Glycostem Therapeutics BV	I/II	December-20	April-23	
NCT05334693	Expanded Haploidentical Natural Killer Cells as Consolidation Strategy for Children/Young Adults With AML	RECRUITING	The purpose of this study is to estimate the efficacy of immunotherapy with ex vivo expanded haploidentical NK cells as consolidation therapy for children/young adults with intermediate risk AML.	AML	Belarusian Research Center for Pediatric Oncology, Hematology and Immunology	I/II	November-21	June-26	
NCT04347616	Natural Killer-cell Therapy for Acute Myeloid Leukemia	RECRUITING	Administration of ex vivo-generated allogeneic natural killer (NK) cells with preceding non-myeloablative conditioning chemotherapy with or without subsequent *in vivo* IL-2 cytokine support.	AML	Radboud University Medical Center	I/II	December-20	September-23	
NCT05272293	Immunotherapy With ex Vivo Expanded Haploidentical Natural Killer Cells for Children/Young Adults With AML	RECRUITING	The purpose of this study is to estimate the efficacy of immunotherapy with ex vivo expanded haploidentical NK cells for children/young adults with primary high risk or refractory AML and relapsed AML.	AML	Belarusian Research Center for Pediatric Oncology, Hematology and Immunology	I/II	November-21	June-26	
NCT05665114	Natural Killer(NK) Cell Therapy in r/r AML	RECRUITING	This is an open-label, Phase I study of QN-030a (allogeneic NK cell therapy) in relapse/refractory Acute Myeloid Leukemia (AML). This clinical study is to evaluate the safety, tolerability and preliminary efficacy of QN-030a in patients with r/r AML,.	AML	Zhejiang University	I	December-22	December-25	
NCT03068819	Cytokine Induced Memory-like NK Cell Adoptive Therapy for Relapsed AML After Allogeneic Hematopoietic Cell Transplant	RECRUITING	Testing the ability of cytokine-induced memory-like (CIML) natural killer (NK) cells in combination with donor-lymphocyte infusion (DLI) to enhance the graft versus leukemia effect in myeloid leukemic children relapsed after transplant.	AML	Washington University School of Medicine	I/II	October-17	October-26	
NCT05601466	Natural Killer(NK) Cell Therapy for Acute Myeloid Leukemia	RECRUITING	This is an open-label, Phase I study of QN-023a (allogeneic CAR-NK cells targeting CD33) in relapsed/refractory Acute Myeloid Leukemia (AML).	AML	Institute of Hematology & Blood Diseases Hospital	I	October-22	October-25	
NCT05400122	Natural Killer (NK) Cells in Combination With Interleukin-2 (IL-2) and Transforming Growth Factor Beta (TGFbeta) Receptor I Inhibitor Vactosertib in Cancer	RECRUITING	Test the hypothesis that combination of two medicines (vactosertib and IL-2) with NK cells will be safe and will activate the donor NK cells. NK cells and vactosertib are experimental because they are not approved by the Food and Drug Administration (FDA). IL-2 (Proleukin¬Æ) has been approved by the FDA for treating other cancers, but the doses used in this study are lower than the approved doses and it is not approved to treat colorectal cancer or blood cancers.	Myeloid malignancies and colorectal cancer	Jennifer Eva Selfridge	I	September-22	December-24	
NCT02727803	Personalized NK Cell Therapy in CBT	RECRUITING	Adoptive therapy with cord blood-derived NK cells for HLA-C2/C2 patients after cord blood transplantation.	Hematologic malignancies after cord blood transplant	M.D. Anderson Cancer Center	II	May-16	May-25	
NCT05665075	Natural Killer (NK) Cell Therapy Targeting CD33 in Acute Myeloid Leukemia	RECRUITING	This is an open-label, Phase I study of QN-023a (allogeneic CAR-NK cells targeting CD33) in relapsed/refractory Acute Myeloid Leukemia (AML). The clinical study is to evaluate the safety, tolerability and preliminary efficacy of QN-023a in patients with relapsed/refractory AML, where a “3 + 3” enrollment schema will be utilized at dose escalation stage. Up to 19 patients will be enrolled.	AML	Zhejiang University	I	December-22	December-25	
NCT04310592	Natural Killer Cell (CYNK-001) Infusions in Adults With AML	RECRUITING	This study will find the maximum tolerated dose or the maximum planned dose of CYNK-001 which contains natural killer (NK) cells derived from human placental CD34+ cells and culture-expanded.	Myeloid leukemias	Celularity Incorporated	I	March-20	December-24	
NCT05115630	Off-the-shelf NK Cells + SCT for Myeloid Malignancies	RECRUITING	Evaluate the safety and effectiveness of giving KDS-1001, an allogeneic NK product, in combination with a standard stem cell transplant to patients with acute myeloid leukemia (AML), myelodysplastic syndrome (MDS), or chronic myeloid leukemia (CML).	Myeloid malignancies	M.D. Anderson Cancer Center	I/II	April-22	June-24	
NCT05092451	Phase I/II Study of CAR.70- Engineered IL15-transduced Cord Blood-derived NK Cells in Conjunction With Lymphodepleting Chemotherapy for the Management of Relapse/Refractory Hematological Malignances	RECRUITING	Evaluate the safety of giving immune cells called natural killer (NK) cells with chemotherapy to patients with leukemia, lymphoma, or multiple myeloma.	B-Cell Lymphoma, MDS/AML	M.D. Anderson Cancer Center	I/II	November-22	August-23	
NCT03300492	Expanded Natural Killer Cells Following Haploidentical HSCT for AML/MDS	RECRUITING	Evaluate safety and efficacy of expanded natural killer cells (NK cells) following haploidentical allogeneic hematopoietic stem cell transplantation (haplo-HSCT) for AML or MDS. Study participants undergoing haplo-HSCT will receive expanded NK cells from their respective stem-cell donors following haplo-HSCT.	MDS/AML	University Hospital, Basel, Switzerland	I/II	November-18	January-23	
NCT05601830	Natural Killer(NK) Cell Therapy for AML Minimal Residual Disease	RECRUITING	Evaluate the safety, tolerability and preliminary efficacy of QN-020a in patients with AML MRD, where a “3 + 3” enrollment schema will be utilized at dose escalation stage. Up to 18 patients will be enrolled.	AML with MRD post-transplant	Institute of Hematology & Blood Diseases Hospital	I	October-22	October-25	
NCT02782546	Cytokine Induced Memory-like NK Cell Adoptive Therapy After Haploidentical Donor Hematopoietic Cell Transplantation	RECRUITING	Demonstrate improvement in the 100 day leukemia free survival to 30% from <10% expected with the use of reduced intensity haplo-HCT in this extremely high-risk patient cohort (based on the institutional experience using non-myeloablative/reduced intensity conditioning in a similar patient cohort).	AML	Washington University School of Medicine	II	January-17	January-25	
NCT05712278	A Study to Investigate Use of Off-the-shelf Natural Killer (NK) Cells (SAR445419) in Relapsed/Refractory Acute Myeloid Leukemia	RECRUITING	Evaluate safety, tolerability, and preliminary anti-tumor activity of SAR445419 administered after fludarabine and cytarabine conditioning for the treatment of relapsed or refractory adult acute myeloid leukemia (R/R AML).	AML	Sanofi	I	June-23	February-25	
NCT05503134	Safety and Efficacy of Expanded, Universal Donor Natural Killer Cells for Relapsed/Refractory AML	RECRUITING	This is a phase I/II dose escalation study designed to determine the safety and estimate the efficacy of UD-NK cells combined with FLA chemotherapy in patients age 18-24.99 with relapsed or refractory acute myeloid leukemia.	AML	Nationwide Children’s Hospital	I/II	February-22	February-27	
NCT04220684	Ph1 Trial Test Safety of IL-21 NK Cells for Induction of R/R AML	RECRUITING	Evaluate the side effects of donor natural killer (NK) cell therapy in treating patients with relapsed/refractory acute myeloid leukemia that has come back (recurrent) or has not responded to treatment (refractory).	AML	Sumithira Vasu	I	June-20	December-22	
NCT05580601	Cytokine-Induced Memory-Like Natural Killer Cells (CIML-NK) for Relapsed & Refractory Acute Myeloid Leukemia (AML)	RECRUITING	Evaluate generation of cytokine-induced memory-like natural killer cells safe infusion into patients with relapsed or refractory acute myeloid leukemia (AML). A secondary objective is to assess efficacy of the CIML-NK cells in treating AML.	AML	Children’s Hospital Medical Center, Cincinnati	I/II	May-23	July-26	
NCT05744440	Safety and Efficacy of Allogenic NK Cells in Combination With Chemotherapy in the Treatment of r/r AML After Allo-HSCT	RECRUITING	Evaluate efficacy and safety of allogenic NK cells in subjects with refractory or relapsed AML after allogeneic hematopoietic stem cell transplantation.	AML	Xuzhou Medical University	I	March-23	May-25	
NCT04623944	NKX101, Intravenous Allogeneic CAR NK Cells, in Adults With AML or MDS	RECRUITING	This is a single arm, open-label, multi-center, Phase 1 study to determine safety and tolerability of an experimental therapy called NKX101 (allogeneic CAR NK cells targeting NKG2D ligands) in patients with relapsed/refractory AML or intermediate, high and very high risk relapsed/refractory MDS	MDS/AML	Nkarta Inc	I	September-20	July-23	
NCT04221971	NK Cell Infusion for Patients With Acute Myeloid Leukemia	UNKNOWN	Evaluate the safety and efficacy of chemotherapy combined with donor-derived *in vitro* activated NK cells infusion for high risk AML patients.	AML	Peking University People’s Hospital	I	October-20	April-22	
NCT04209712	Natural Killer Cells Infusion for Treating Acute Myeloid Leukemia Patients With Minimal Residual Disease	UNKNOWN	This trial will evaluate the effectiveness and safety of haploid donor-derived *in vitro* activated NK cells infusion for treating acute myeloid leukemia patients with minimal residual disease.	AML	Shanghai iCELL Biotechnology Co., Ltd, Shanghai, China	I	January-20	December-21	
NCT03955848	Infusion of alloreactive NK cells as consolidation strategy for AML patients	UNKNOWN	Using a genetic randomization through a ‘donor’ vs ‘no donor’ approach, patients will undergo KIR-mismatched NK cell infusion (ARM 1) or followed-up without treatment (ARM 2).	AML	IRCCS Azienda Ospedaliero-Universitaria di Bologna	III	May-18	May-22	
NCT02809092	Interleukin-21 (IL-21)- Expanded Natural Killer Cells for Induction of Acute Myeloid Leukemia	UNKNOWN	Determine the feasibility and maximum tolerated dose of expanded NK cells and estimate the toxicity of treating relapsed/refractory AML with fludarabine + high-dose cytarabine + G-CSF (FLAG) chemotherapy followed by haploidentical expanded natural killer (NK) cells.	AML	Hospital de Clinicas de Porto Alegre	I/II	April-17	September-20	
NCT01619761	NK Cells in Cord Blood Transplantation	UNKNOWN	Evaluate the side effects and best way to give NK cells and donor umbilical cord blood transplant in treating patients with hematological malignancies.	Myeloid malignancies	M.D. Anderson Cancer Center	I	May-13	November-21	
NCT01520558	CNDO-109-AANK for AML in First Complete Remission (CR1)	UNKNOWN	Examine the safety of infusing escalating doses of CNDO-109-Activated Allogeneic NK Cells, after a preparatory chemotherapy regimen, in adult patients with AML who are in their first complete remission at the time of enrollment, are not candidates for stem cell transplant, and are considered to be at high risk for recurrence.	AML	Coronado Biosciences, Inc.	I/II	December-12	February-18	
NCT00799799	Adoptive Immunotherapy of High Risk Acute Myeloblastic Leukemia Patients Using Haploidentical Kir Ligand-mismatched Natural Killer Cells	UNKNOWN	Evaluate benefit of haploidentical NK cell infusion in AML patients with *de-novo* or secondary disease with age greater than 18 years not eligible for stem cell transplantation for medical contraindications, lack of donor, or lack of stem cells.	Myeloblastic Leukemia	University of Bologna	I	October-05	December-09	
NCT05886491	A Study of GDX012 in Adults With Relapsed or Refractory Acute Myeloid Leukemia	NOT_YET_ RECRUITING	Determine safety, optimal dosing and tolerability of GDX012, a non-engineered healthy-donor derived yd T cell therapy, in relapsed/refractory AML patients	AML	Takeda	I/II	July-23	June-27	
NCT03533816	Expanded/Activated Gamma Delta T-cell Infusion Following Hematopoietic Stem Cell Transplantation and Post-transplant Cyclophosphamide	RECRUITING	This study uses gamma delta T-cells post- haploidentical transpolant to maximize the anti-tumor response and minimize graft versus host disease (GVHD) in leukemic and myelodysplastic patients.	Myeloid malignancies	University of Kansas Medical Center	I	January-20	January-25	
NCT05015426	Gamma Delta T-cell Infusion for AML at High Risk of Relapse After Allo HCT	RECRUITING	Evaluate the maximum tolerated dose (MTD) and effectiveness of Artificial Antigen Presenting Cell (AAPC)-expanded donor T-cells administered as a single infusion after an allogeneic hematopoietic cell transplant (alloHCT) to treat patients with Acute Myeloid Leukemia (AML).	AML	H. Lee Moffitt Cancer Center and Research Institute	I	March-22	December-24	
NCT04008381	Ex-vivo Expanded Gamma Delta T Lymphocytes in Patients With Refractory/Relapsed Acute Myeloid Leukaemia	UNKNOWN	Evaluate the safety and efficacy of ex-vivo expanded γδ T-lymphocytes in patients with relapsed or refractory acute myeloid leukemia. PBMCs will be separated from peripheral blood of suitable donors. After making them potential cancer killer γδ T Cells, they will be infused to the patients as an immunotherapy treatment.	AML	Wuhan Union Hospital, China	I	September-19	January-23	

Shading in table cells differentiate cell types.

## αβT cells in AML

### αβT cell biology

αβT cells recognize their cellular targets through a well-characterized interaction of peptides (a.k.a. antigens) presented by classical MHC molecules binding to the hyper-variable regions of cognate heterodimeric α and β TCR chains (**Figure 1A**) (8). αβT cells are educated in the thymus to recognize foreign or neo-self-antigens, but spare self-antigens to prevent autoimmunity. Their TCR genes undergo somatic hypermutation of V, D, and J segments resulting in a limitless array of TCR affinities. The thymic education process involves presentation of self-peptides by thymic epithelial cells on MHC. T cell clones with high affinity for self-peptide-MHC complexes are deleted to prevent autoimmunity. Those with no affinity for self-MHC are also deleted through lack of TCR signaling and neglect (9). It is estimated that only about 2% of all T cell clones generated are able to successfully complete thymic selection process (10). Developing T cell progenitors pass through a phase of double-positivity with expression of both CD4 and CD8. Guided by their TCR affinity for MHC class I or class II (MHC-I or MHC-II, respectively), they then segregate into the two canonical single-positive T cell lineages and maintain expression of just CD4 or CD8.

CD4 T cells recognize peptide-MHC-II complexes present on professional antigen-presenting cells (APCs) of hematopoietic origin and provide help in the form of cytokines to modulate the inflammatory milieu and orchestrate appropriate immune responses for each context. Based on the type of response required, they are classified into subsets including Th1, Th2, Th17, and regulatory T cells (Tregs). Th1 CD4 T cells render the classical inflammatory state with high interferon gamma (IFNγ) and tumor necrosis factor alpha (TNFα) production and are crucial in immune responses to viruses and bacteria. Th2s produce more interleukin(IL)-4, IL-5 and IL-13 and are more important in helping B cell antibody production to fight against extracellular parasites and bacteria. Th17s produce more IL-17 and play roles in a variety of contexts including mucosal barrier protection and autoimmunity. Tregs are regulatory CD4 T cells that produce anti-inflammatory cytokines that help reduce inflammation and promote tissue repair. It was first thought these different CD4 T cell subsets represented terminal differentiation states, but evidence has shown some interconversion in specific contexts, with inflammatory T cells acquiring some regulatory functions in settings of chronic inflammation (11).

CD8 T cells recognize peptide-MHC-I complexes that are more ubiquitously expressed in all tissues and nucleated cells except red blood cells, and when provided appropriate co-stimulation and cytokine activation, can become highly cytotoxic. Thus CD8 T cells are thought to be a main effector cell population in many immune responses and can kill any MHC-I expressing target cell when fully activated. Additionally, after a well-orchestrated activation phase, CD8 T cells expand rapidly to create a pool of clones with the same specificity with cytolytic capacity. Many of these clones die towards the end of an immune response, while some convert to a memory phenotype, which are long-lived and help mount even more effective immune responses upon re-exposure to their antigens (e.g. upon reinfection with a particular virus or bacteria).

### Strengths and limitations of αβT cells as adoptive cell therapy for AML

Potent cytotoxicity, the ability to respond to an extensive array of antigens, expand exponentially and induce long-lived memory T cells which are capable of mounting a prompt immune response, are all at-first-glance desirable properties for a potential cell therapy (12). Indeed, all currently FDA approved CAR-T designs use αβT cells as their primary cell substrate (13). In AML, the potentially curative GVL effect of an allogeneic transplant and any benefit of the relatively rudimentary approach of DLIs after transplant is thought to rely on αβT cell clones reactive to residual leukemic cells. Research on improving transplant outcomes has focused on increasing the donor pool through cord blood and haplo-transplant approaches, as well as strategies to prevent GVHD. Haploidentical transplants have improved dramatically in recent years and have the potential for increased GVL effects due to partial HLA mismatching (14). Strategies to improve DLIs include CD4 or CD8 clone selection and ex vivo activation. For example, in a small sample of 4 patients, a CD4 purified DLI was shown to selectively mediate GVL effects without inducing GVHD by recognizing miHA on MHC-II molecules selectively expressed in host hematopoietic cells (15). In mouse models, DLIs consisting of enriched memory CD4 or CD8 T cells could eradicate leukemic cells without inducing GVHD (16, 17). A memory CD8-enriched DLI was tested in 15 patients and was shown to be safe, but anti-leukemic efficacy remains to be proven (18). In a phase 1 trial of DLI activated ex vivo with anti-CD3/CD28 co-stimulation in 18 patients with aggressive malignancies relapsing after transplant, 2 of 4 AML patients included achieved complete remissions (19). Thus improvements on the historical strategies of allogeneic transplant and DLIs are being made for AML patients and rely on αβT cell anti-leukemic immune functions. αβT cell-based cell therapies for AML in the non-transplant setting have used CAR- or TCR-engineered cell products. Many groups are developing anti-AML CAR designs with targets including CD33, folate receptor β (FRβ), CD123, C-type lectin molecule 1 (CLL1), PR1/HLA-A2, CD70, T cell immunoglobulin mucin-3 (TIM-3), CD13, and CD93. TCR-engineered targets include Wilm’s tumor 1 (WT1), Surviving, minor histocompatibility antigen HA1, telomerase (TERT), mutated Nucleophosmin1 (NPM1), murine double minute 2 (MDM2), and others. Results of preclinical and early clinical trials with these CAR- and TCR-engineered T cells in AML have been extensively reviewed elsewhere (20, 21). While preliminary results vary, CAR and TCR-engineered T cell therapies for AML are progressing towards improved specificity to leukemic targets, decreased toxicity against normal hematopoietic cells, prolonged persistence, and resistance to exhaustion.

Several limitations to αβT cell biology have hindered success as a primary cell therapy in AML. First, the antigen-dependent activation of αβT cells becomes ineffective once their neoplastic targets stop expressing the target antigen or loading and presenting it on MHC. Several studies have demonstrated MHC-II downregulation in relapsed AML after transplant, implicating decreased antigen presentation as a means of immune escape from GVL effects (22, 23). CAR T cells’ synthetic receptors are one means to circumvent MHC-dependent antigen presentation, but CARs are still highly restricted and dependent on specific cell-surface protein expression, thus still susceptible to antigen loss. Loss of CD19 has been reported as a common mechanism for relapse in B cell malignancies after CD19-directed CAR therapy (24, 25). Similar escape via antigen-loss will likely occur in AML-directed CARs. Identification of antigens critical for cell survival will help prevent this type of relapse.

Second, immune-activating and truly myeloid leukemia-specific targets have yet to be identified. Most AML antigens and surface-proteins explored are also expressed on normal hematopoietic stem and progenitor cells, thus their destruction leads to catastrophic aplasia or hematologic deficiencies (26). For instance, anti-CD33 directed CARs result in significant non-malignant myeloid toxicity. This led Kenderian et al. to develop a transient mRNA-based anti-CD33 CAR expression platform that could be used to eliminate the bulk of AML but allow for subsequent hematopoietic recovery (27). In their approach, a single-chain variable fragment (scFv) derived from the clinically approved gentuzumab ozogamicin antibody-drug conjugate was used as the receptor component of a CAR. Electroporation of T cells with this anti-CD33-CAR efficiently eliminated CD33+ AML cells, but subsequently decreased transgene expression permitted regrowth of normal myeloid cells. Fine-tuning CAR sensitivities or utilizing bi-specific CAR designs could further help improve AML killing while sparing healthy hematopoietic cells. Logic-gated CAR designs that require simultaneous engagement by two distinct antigens, or the absence of a third antigen, could be exploited to specifically recognize unique expression patterns of antigens on leukemic stem cells or AML cells vs normal hematopoietic progenitors (28, 29).

Third, due to their self-MHC restriction, αβT cells do not function properly in MHC/human leukocyte antigen (HLA)-mismatched individuals. While some degree of mismatch is essential for GVL effects post-transplant, it is the same mismatch that mediates GVHD. Mismatch allows for donor T cells to recognize the recipient’s cells as foreign. Too little mismatch, and the donor T cells cannot attack the host’s leukemia. Too much mismatch, and all the host’s cells, including normal tissue cells, appear foreign resulting in rampant destruction of normal host organs. The potential for dysfunction, rejection, or toxicity due to HLA mismatch currently restricts engineered αβT cell adoptive therapies to autologous applications. Persistent expression of non-specific endogenous TCRs in CAR-T products raises the concern for unwanted off-leukemic antigen activation. In the case of engineered-TCRs, pair in alpha and beta-chain combinations with unknown TCR specificities. GVHD induction has been observed in post-transplant patients receiving donor-derived CAR-T cells for B cell malignancies (30, 31). Groups are attempting to circumvent these issues by knocking out endogenous TCRs in engineered products. For example, the UCART7 product for CD7+ acute lymphocytic leukemia (ALL) uses clustered regularly interspaced palindromic repeat (CRISPR)/Cas9 editing to simultaneously eliminate the endogenous T cell receptor alpha constant (*TRAC*) and *CD7* genes, preventing GVHD and fratricide, respectively (32). In another approach, Michaux et al. engineered a novel truncated CD3ζ molecule that incorporates into and interferes with endogenous TCR signaling and prevented GVHD in mouse models (33). While promising, all these advanced engineering approaches increase the complexity of the engineering process and require further laborious and time-consuming *ex vivo* manipulation (34).

Finally, upon activation, conventional T cells upregulate a variety of inhibitory receptors and over-time become highly susceptible to exhaustion and anergy (35). In some contexts αβT cells can even differentiate into Tregs, which then can actively inhibit anti-tumor immune responses (36). These anti-inflammatory or T cell inhibitory mechanisms exist to prevent unwanted chronic inflammation resulting from natural immune responses. However, in the context of cancer, the anti-inflammatory TME results in inhibition of anti-tumor T cell responses and permits immune escape. The flexibility of T cell phenotypes helps them respond appropriately to different threats but renders them susceptible to inhibition by immunosuppressive malignant clones. Intrinsic immunosuppression underlies the efficacy of the checkpoint inhibitors programmed cell death protein 1 (PD-1) and cytotoxic T-lymphocyte associated protein 4 (CTLA-4) in many cancer types (37). Programmed death-ligand 1 (PDL-1) has been shown to be increased on AML blasts at relapse (23), PD-1 high T cell phenotypes predict relapse after transplant (38), and PD-1 checkpoint blockade has shown some promise in treating AML patients who relapsed after transplant (39). AML has also been shown to inhibit αβT cell responses and promote Treg and myeloid-derived suppressor cell phenotypes through production of high levels of soluble factors like reactive oxygen species (ROS), and indoleamine 2,3-dioxygenase (IDO) (40, 41). Strategies to prevent exhaustion of αβT adoptive cell therapies and promote persistent activated states will be key to their success.

In conclusion, while the cytotoxicity and expansion of T cells are favorable qualities, their specificity to HLA alleles, potential for GVHD induction, lack of AML-specific targets, and propensity for exhaustion are all hurdles that prevent the broad use and limit the efficacy of αβT cells for therapies in AML.

## NK cells in AML

### NK cell biology

NK cells are the best studied cytotoxic lymphoid subset other than αβT cells in AML, and are the only cytotoxic cell type amongst the innate lymphoid cell (ILC) arm of the immune system (42). Distinct from T cells whose maturation occurs largely in the thymus, NK cells develop and mature primarily in the bone marrow and secondary lymphoid organs. At steady state, they represent 5-20% of all circulating peripheral blood mononuclear cells (PBMCs), and can be found as resident cells in many lymphoid and non-lymphoid organs including the bone marrow (43). NK cells share effector functions with conventional αβT cells, particularly CD8 cytotoxic T lymphocytes (CTLs). Upon activation, NK cells secrete significant amounts of IFNγ,TNFα, IL-10 and other pro-inflammatory cytokines, and use granzyme B and perforins to destroy target cells. However, their means of differentiating a cancer cell from healthy cells is distinct from T cells. They do not have a TCR or other polymorphic receptor that undergoes somatic mutation for wide antigen-recognition diversity (thus their consideration as part of the innate rather than adaptive immune system). Instead, NK cell activity is regulated by an array of conserved activating and inhibitory receptors, the balance of which results in inflammatory/cytotoxic versus inhibitory functions (**Figure 1B**).

One of the best-studied activating receptors is NKG2D, a potent activating receptor which binds to MICA/B and ULBP-family molecules. NKG2D ligands are upregulated on a diversity of stressed and neoplastic cells through mechanisms involving oxidative stress, DNA damage, and inflammatory cytokine exposure (44). NKG2D can also be expressed on αβ and γδT cells, where it serves as a costimulatory molecule augmenting TCR signaling (45). Other important activating receptors include NKp30 (ligands B7-H6 and BAT3), LFA-1 (ligand (ICAM-1), CD27 (ligand CD70), and DNAM-1 (aka CD226, ligands CD155 and CD112), CD16 (an immunoglobulin Fc receptor), and CRTAM (ligand Necl-2) (46). Inhibitory receptors on NK cells include pan-lymphocyte receptors such as PD-1, TIM3, lymphocyte-activation gene 3 (LAG3), and T cell immunoglobulin and ITIM domain (TIGIT), as well as a panel of unique inhibitory killer Ig-like receptors (KIRs) that bind to HLA-A, B, and C molecules (47). KIRs are more diverse and individualized than other inhibitory NK receptors, where the repertoire of KIRs matches a person’s HLA allelic profile. The interaction of specific HLA types with matched KIRs on NK cells is a means of self-recognition and helps prevent autoimmune destruction. HLA-E is a non-canonical MHC that presents the signal peptides from the other conventional MHC-I molecules. Binding of HLA-E to NKG2A/CD94 heterodimers on NK cells (as well as other lymphocyte subsets), also inhibits NK activation (48). Loss of HLA and thereby loss of antigen presentation is a common strategy for viral and tumor escape from CTL destruction. However, this same loss (a.k.a. “missing-self”) results in a decrease in inhibitory receptor engagement on NK cells, thus allowing for activation and destruction (49). It is thought that NK cells evolved missing-self recognition and their complementary array of activating and inhibitory receptors to constantly survey for, recognize, and destroy infected and neoplastic cells. NK cells thereby represent an important intrinsic defense mechanism against cancer development throughout the body.

Besides the lack of thymic-educated polymorphic antigen-recognition machinery, NK cells exhibit several significant differences from T cells. They circulate at constitutive basal levels, and while they do expand in response to inflammatory conditions, particularly viral infections, this a non-clonal 10-100s fold expansion compared to the individual and 1000s-fold clonal expansion and differentiation that occurs in T cells (50). Second, most conventional NK cells have a much shorter lifespan than T cells, on the order of several days to weeks (51). While some “adaptive” or memory-like NK cells can develop in response to viral infections and IL-12/15/18 cytokine cocktails and be long-lived (>6 months), their ability to generate a true antigen-specific memory response is not well understood (52). Third, while unique NK subsets have been characterized, they display heightened plasticity and their phenotype is more dependent upon the immediate milieu of microenvironmental queues rather than a terminal differentiation program (47). For instance, CD56^high^ NK cells are generally less cytotoxic and produce greater amounts of pro-inflammatory cytokines, while CD56^low^ NK cells are more cytotoxic, and exhibit higher chemokine receptor expression for homing to inflamed tissues. Despite their distinct differences, these two subsets are thought to exist along a continuum dependent on the degree of systemic and local inflammation, and CD56^high^ NKs can convert to CD56^low^ NKs in the context of infections, tissue damage, or transformation. No NK subtypes have been identified that are analogous to the distinct phylogeny and roles of Th1, Th2, Th17, or Treg cells. Fourth, while T cells require matched HLA to recognize their antigens and respond appropriately, NK receptors are non-polymorphic and therefore conceivably functional within any recipient in adoptive cell therapies. Subtle differences between host and donor NK phenotypes may in fact be beneficial, as discussed momentarily. For this reason, NK cells are often described as a potential “universal” or “off-the-shelf” therapy, circumventing many of the biologically limiting, logistical, and costly barriers inherent to T cell based adoptive approaches. Finally, exhausted, senescent and anergic T cell phenotypes after chronic stimulation are well-defined and more terminal. While dysfunctional NK cell phenotypes can exist and are sometimes defined as exhausted or anergic due to decreased functionality, these definitions and their permanence in NK cells are still highly debated topics (53, 54).

### NK cell status in AML patients – intrinsic anti-tumor effects, dysfunction, and under-appreciated roles in conventional therapies

Due to their inherent ability to recognize and destroy transformed cells, NK cells exert a significant selective pressure on tumor development. For this reason, NK-resistant and suppressive tumor cell phenotypes and NK dysfunction are often observed in overt malignancy. This is particularly true in AML (55, 56). Compared to CD8 T and γδT cells, NK cell function was the most negatively impacted at AML diagnosis, with decreased cytotoxicity and durably impaired pro-inflammatory cytokine production (57). NK cells from AML patients consistently exhibit decreased cytotoxic receptor expression (particularly NKp30, NKp44, NKp46, and DNAM-1) and decreased cytotoxicity compared to NK cells from healthy donors (58–60). Furthermore, decreased activating NK receptor expression correlates with worse prognosis and inferior outcomes after allo-SCT (61, 62). Stringaris et al. also demonstrated increased expression of the inhibitory receptor NKG2A in AML patients, and showed overexpression of NKG2A in NK cells correlated with failure to achieve remission, as well as impaired cytotoxicity against autologous blasts and leukemic cell lines (63). In a complementary manner, AML cells tend to express lower levels of activating NK ligands, such as ULBP1, ULBP2, and ULBP3, compared to normal hematopoietic cells (64). Pende et al. demonstrated consistent expression of CD155 and CD112 on human leukemic samples, ligands for the activating NK receptor CD226 as well as the inhibitory receptor TIGIT, and correlated the number of NK cells expressing these and other non-HLA-dependent KIRs with the degree of cytotoxicity against leukemic cells (65). This early study did not assess TIGIT expression, but more recent studies have shown increased TIGIT expression by NK cells in AML corresponds to a dysfunctional phenotype and poor prognosis (66), suggesting upregulation of the TIGIT-CD155/CD112 axis may be a key mechanism of AML escape from NK cytotoxicity. Bone marrow resident NK cells exhibit a unique immunosuppressive phenotype with higher surface CXCR6 and express higher levels of TIGIT than circulating NK cells at steady state, suggesting anti-TIGIT approaches may be particularly important for treatment of hematologic malignancies (67). Together, these results consistently demonstrate defective NK phenotypes in AML patients, and NK-suppressive or evasive phenotypes in the leukemic cells themselves, suggesting NK-AML interactions are an important selective pressure in leukemogenesis.

Despite these defects, several studies have demonstrated contributions of NK cells to the success of conventional AML therapies, both chemotherapy and transplants. Regarding chemotherapies, Lowdell et al. showed that *in vitro* leukemia cytolytic activity (LCA) was dependent on CD56+/CD8a+/CD3- NK cells (68). In 25 patients who underwent chemotherapy treatment for acute leukemia, a low or absent LCA was highly predictive of relapse within 2 years (p<0.001). In a flow cytometry-based analysis of 130 AML samples, increased frequency of NK cells (>5%) was found to be an independent predictor of improved overall and disease-free survival in response to standard of care treatment (69). Additionally, degree of NK cytotoxicity was a predictor of overall survival in response to the hypomethylating agent azacytidine (70). Together, these findings have led to the hypothesis that NK activity as opposed to direct effects of chemotherapy were responsible for durable remissions in AML patients, or that certain standard-of-care treatments work in part by potentiating anti-leukemic NK responses.

Regarding the role of NK cells in transplant settings, Chretien et al. found that the high activating NKp46 expression at diagnosis correlated with improved overall survival after allo-SCT. This suggests a more activated phenotype of host NKs in some way augments the benefit of a transplant, although the mechanistic basis for this is unclear (71). A more robust positive effect on outcomes has been established for rapid donor NK reconstitution (72–74). It is thought the improved outcomes reflect increased NK-mediated GVL effects; however, NK engraftment could just reflect improved engraftment overall or could be dependent on differences in donor sources or other variables that also improve outcomes. Indeed, rapid NK engraftment is particularly accentuated after cord blood transplants, thus may have a larger impact in this setting (72).

KIR-HLA mismatch is another major contributor to NK-mediated effects after transplant. HLA mismatch (or incompatibility) prevents engagement of inhibitory KIR on donor NKs by certain HLA-alleles of the host, allowing donor-NKs to destroy residual host hematopoietic cells through “missing self” mechanisms. In a pioneering study, Ruggeri et al. retrospectively analyzed 57 post-transplant AML patients for KIR-HLA mismatch (75). 5 year event free survival rate in recipients of KIR-HLA incompatible transplants was 60%, compared to 5% in KIR-HLA compatible recipients (p<0.0005), suggesting an enormous impact of NK-mediated killing of AML in an HLA-mismatch dependent manner. The effect of KIR-HLA mismatch on transplant outcomes in AML is an ongoing area of intense research (76–78).

Finally, mounting conflicting evidence has revealed a complex relationship between NK cells and the development of GVHD after SCTs (79). Early pathologic studies of organs affected by GVHD demonstrated infiltration by donor-derived NKs, raising the concern that NKs were part of the cause of GVHD (80). However, many subsequent reports in mouse models and humans showed that alloreactive NK cells could actually prevent GHVD development by depleting host-antigen-presenting cells and thereby prevent host-reactive T cell activation, or by direct cytotoxicity against activated T cells (75, 81–86). Furthermore, infusion of enriched donor NK cells into transplant recipients reduced the incidence of GVHD (87), and NK cells activated *ex vivo* with IL-12, IL-15, and IL-18 (which induces a more memory-like phenotype as discussed below) helped prevent GVHD in a mouse model of HLA-mismatched SCT (88). Conflicting with this data, some modern studies in mouse models and humans have shown an increase in GVHD-incidence or causation as a result of NK cells, thought due to inflammatory cytokine (IFNγ and TNFα) production by the NK cells driving GVHD-T cell responses (89–91). In the study by Shah et al. (90), the NK cells had been activated *ex vivo* using IL-15 and 41BBL, thus may have been in a more proinflammatory state when infused into the recipients. Altogether this data supports a potential role for NK cells in suppressing GVHD, however careful selection of clinical context and NK activation state is crucial to prevent the opposite effect.

### NK cells as adoptive cell therapy for AML

Given their inherent involvement in AML recognition, and the identification of defective NK phenotypes in leukemic patients, many adoptive NK strategies are under development to boost recognition of, and cytotoxicity against AML. These strategies include using unmanipulated NK infusions that rely on their inherent anti-leukemic effects, conditioning NKs with drugs or cytokines to boost their activation and persistence, combining NK therapies with checkpoint blockade to avoid their suppression, and finally engineering NK cells like CAR-T therapies. **Table 2** provides a list of all ongoing and completed phase II and III clinical trials using these cell types as therapies for treating AML.

Early NK-based strategies only used *ex vivo* IL-2-activation prior to infusion to help treat relapsed disease, and despite testing in high-risk refractory and relapsed patients still showed promising results. In a preliminary study by Miller et al. in 2005, 19 AML patients with poor prognosis were treated with allogeneic haploidentical donor NK cells (92). The only pre-transplant treatment of the NKs was magnetic bead-based CD3 depletion and overnight incubation with IL-2. A high-intensity conditioning regimen of cyclophosphamide and fludarabine was administered 48 hours before cell infusion, and post-transplant patients received ongoing subcutaneous IL-2 injections. NK infusion resulted in complete hematologic remissions in 5 of 19 patients (26.3%), many of whom were refractory to multiple lines of treatment or had relapsed after transplant. Follow-up studies have tried to better understand why some patients respond while others do not, with the goal of improving response rates through changes in ex vivo manipulation of the NK product. Curti et al. followed the same overall treatment strategy in patients in first complete remission, and found a correlation between the number of allogeneic NKs infused and disease-free survival duration (93). In an attempt to avoid increased GVHD rates with haploidentical transplants yet still benefit from KIR-HLA mismatched NK cells, Lee et al. infused varying numbers of haplo-identical NK cells from a first donor prior to an HLA-matched transplant from a second donor. Despite small sample size, they found a significant trend towards improved survival in NK-treated patients (91). More recently, Bjorklund et al. transfused IL-2-activated haploidentical NK cells after cyclophosphamide and fludarabine conditioning and total lymphoid irradiation in relapsed/refractory myelodysplastic syndrome (MDS) and AML patients, and did not use subcutaneous IL-2 post-infusion (94). 6 of 16 patients treated achieved (37.5%) partial or complete remissions, and 5 patients proceeded to transplant. To improve NK persistence and response rates, other groups are currently experimenting with a variety of ex vivo activation protocols. A now-well-established and potent strategy for ex vivo NK expansion is the use of membrane-bound IL-21-expressing feeder cells, which can lead to expand NK cells thousands-fold in just a few weeks’ time without a decrease in cytotoxicity or other effector functions (95, 96). In a phase I/II clinical trial in patients with myeloid malignancies, membrane-bound IL-21 (mbIL-21)-expanded donor-derived NK cells transfused after transplant were associated with greatly improved 2-year relapse rates (4% vs 38% in controls), and did not result in increased toxicity or treatment-related mortality (97). Incubation with a combination of IL-12, IL-15, and IL-18 results in NK cells with a “memory-like” phenotype of improved persistence and cytokine responsiveness. A trial using these cytokine-induced memory-like (ML) NK cells for treatment of relapsed/refractory AML resulted in improved response rates (>50%) compared to historical results with IL-2 activated NK cells (98). In a second trial in pediatric and young adult patients who relapsed after transplant, ML NK cells induced complete remissions in 4 of 8 patients (99). In a third trial, donor-derived ML NK cell infusion followed by an experimental IL-15 superagonist N-803 resulted in a composite complete remission rate of 83% as assessed on day 28 after cell infusion in patients relapsing after transplant (100). Preliminary results from an ongoing trial treating adult patients who relapsed post-transplant with ML NK cells has demonstrated 10-50 fold *in vivo* expansion and persistence over several months of evaluation (101). Another approach to boost NK cytotoxicity is to use tumor cell lysates to provide a priming signal ex vivo prior to NK infusion. CTV-1 leukemia cell-line lysate treated (CNDO-109-activated) NK cells demonstrated increased cytotoxicity against NK-resistant cell lines, and were well-tolerated and induced complete remissions in a significant proportion of patients in phase I trials (102, 103). The optimal pre-infusion expansion/activation, dose, source (autologous, SCT donor-derived, or HLA-mismatched), and timing of treatment (at first remission, prophylactically after transplant, at relapse, etc.), with NK cells are all important areas of ongoing research.

Given their phenotypic dependence on the immunologic microenvironment, another strategy to boost NK-killing of AML is through combination treatment with immunomodulatory agents. Treatment of patients in remission with histamine dihydrochloride and low-dose IL-2 resulted in expansion of NK cells with increased expression of activating receptors NKp30 and NKp46, and correlated with improved leukemia-free and overall survival (104). Aside from direct effects on leukemic cells, hypomethylating agents have been shown to decrease shedding of soluble NKG2D ligands, which reduces chronic ligand-dependent exhaustion of NK cells (105). Presumably through a similar mechanism, concurrent decitabine enhanced anti-CD33 monoclonal antibody mediated cellular cytotoxicity of NK cells against AML blasts (106). The immunomodulatory agents lenalidomide and pomalidomide were similarly shown to potentiate anti-leukemic and anti-MDS NK responses through several mechanisms including increased IL-2 and IL-15 production and increased expression of activating receptors (107, 108). Due to their potent activation by way of the Fc-receptor CD16a, several groups have employed anti-leukemic monoclonal antibodies (mAbs), bispecific killer-engagers (BiKEs, comprised of an anti-CD16 scFv and an anti-leukemic scFvs) (109), and trispecific killer-engagers (TriKEs, which add an IL-15 crosslinker) (110) and shown potent NK-mediated cytotoxicity. A phase I/II trial of the TriKE GTB-3550 has completed accrual (NCT03214666). Targets used in these antibody- or scFv-dependent strategies include CD123 (111), CD133 (112), the IL1RAP (113), and CD33 (110), but unfortunately harbor significant on-target-off-leukemic toxicity due to expression of these markers on normal hematopoietic cells. While these studies focused on immunomodulatory effects on endogenous NK cells, one could envision their potential application in combination with adoptive NK therapies.

Due to the fact that NK cells can express various inhibitory receptors including PD-1, TIM3, Lag3, and TIGIT, and that higher expression of these inhibitory receptors was associated with worse prognosis in AML (66), there is rationale for using inhibitory receptor blockade to bolster NK-mediated immune responses against leukemia. However, few studies have examined the potential of this type of approach. In a preclinical model using the NK-92 cell line, combined TIGIT and CD39 or A2AR blockade did increase cytotoxicity against AML cells *in vitro*, with the authors concluding this approach should be tested clinically (114). Vey et al. conducted a phase I trial of an anti-inhibitory-KIR mAb IPH2101 in 23 AML patients in first complete remission, and found no changes in *in vitro* cytotoxicity or phenotype of NK cells other than transiently increased CD69 expression at the highest dose levels (115). With small sample size and no comparator arm, conclusions about efficacy were only speculative. PD-1 and TIM3 blockade, either alone or in combination with conventional AML therapies like hypomethylating agents, has shown some signal in early clinical trials, but none have examined NK phenotypes or function as clinical correlates, and none combined checkpoint blockade with NK adoptive therapies (116).

A more direct approach to augment NK-based cell therapies is through direct NK engineering. Many pre-clinical studies with NK-based CARs are underway in numerous cancer types, but preclinical and clinical studies are lagging in AML. Based on our understanding of NK biology as described above, putative benefits of NK-based vs T-cell based CARs include decreased risk for rejection in non-HLA matched hosts (thus more universal applicability), potentially decreased GVHD complications depending on the context, and increased potential for NK-AML recognition via endogenous NK receptors particularly in KIR-HLA mismatched settings (117). As with CAR-T cells, killing of normal hematopoietic progenitors or fratricide remain issues for CAR-NK cells in AML. To circumvent this, Gurney et al. engineered anti-CD38 CAR-NK cells with their endogenous CD38 knocked out in order to prevent fratricide (118). In a highly leukemia-specific approach, Dong et al. engineered a CAR- NK cells with specificity against a neoantigen present in NPM1-mutated AML and thus not present in normal hematopoietic cells (119). Their engineered NK cells demonstrated NPM1-mutatant leukemia specific killing, enhanced persistence, and improved overall survival in xenograft models. Clinical studies with CAR-NK cells directed against CD33 and NKG2DL have made the most progress to date. In the case of NKG2DL, the NCT04623944 clinical trial of allogeneic CAR-NK cells in relapse/refractory AML or MDS is still accruing. The anti-CD33 CAR-NK trial was the first-in-human use of the irradiated NK-92 cell line as its adoptive cell substrate. The CAR was comprised of an anti-CD33 scFv fused to CD28 and 41BB signaling domains. Three patients were treated with the first experiencing a potential complete remission after CAR-NK infusion but hematologic relapse 4 months later, and the other two had no meaningful leukemic responses. Despite insufficient outcomes data, this trial demonstrated the feasibility and safety of CAR-NK infusions in AML patients. The benefit of NK-92 cell-line vs primary NK based CARs in AML needs to be further investigated. Furthermore, although the therapy consisted of the NK-92 cell line, these trials highlighted the potential for using KIR-HLA mismatched NK cells for therapeutic purposes.

Aside from engineering antigen-receptors into NK cells, other engineering approaches have been designed to boost NK cell persistence and expansion. Of promise are strategies targeting IL-15, a cytokine known to be important for NK survival. Studies have attempted to boost NK persistence through introducing IL-15 over-expression cassettes in their viral vectors (120, 121). While constitutive IL-15 production is associated with improved NK survival and proliferation, secretion by engineered NK cells was found to be associated with lethal toxicity in one pre-clinical animal model (122). To avoid potential toxicity of systemic IL-15, Zhu et al. knocked out cytokine-inducible sh2-containing protein (CISH), a negative regulator of IL-15 signaling, and showed improved disease control in an AML xenograft model (123). Additionally, Wang et al. created NK cells expressing a rimiducid-inducible MyD88/CD40 (iMC) protein and IL-15 (124). Coupled with a CD123-CAR, the engineered NK cells demonstrated improved inducible NK expansion and cytolytic activity. They further introduced a rapamycin-regulated Caspase-9 (iRC9) gene that served as a safety-switch, allowing for controlled and selective elimination of engineered NK cells. These advanced engineering approaches are early indications of the phenotypic malleability of NK cells via transgene expression, and the potential for NK-based adoptive cell therapies. Many more synthetic-receptor designs harnessing various NK-specific signaling domains and other non-antigen-directed receptors have shown promise in other malignancy types, and could also be utilized in AML (125, 126).

In summary, NK cells are a unique non-polymorphic innate-immune cell type whose cytotoxicity is regulated through a balance of activating and inhibitory receptors. NK cells frequently exhibit dysregulated phenotypes in leukemic patients, suggesting a requirement for NK-immune escape in leukemia. Successful strategies to expand and activate NK cells ex vivo have been developed, and in-human clinical trials with NK adoptive therapies show promising early results. Benefits over αβT cells include more universal off-the-shelf applicability, KIR-HLA mismatch-mediated leukemic cytotoxicity, lack of association with GVHD, and non-MHC dependent activation. CAR-NK development has been hindered in AML due to lack of leukemia-specific targets, but novel engineering approaches informed by CAR-NK design for other malignancies are under development to exploit NK-mediated anti-leukemic responses.

## iNKT cells in AML

### iNKT cell biology

iNKT cells are a nebulous cell type that has both innate and adaptive immune features. Similar to T cells, they develop from CD4/CD8 double-positive precursors in the thymus, but then segregate into their own lineage, defined by their expression of and dependence on the transcription factor promyelotic leukemia zing finger (PLZF) (127). They express unique TCRs that recognize and respond rapidly to lipid antigens presented by the non-classical MHC molecule CD1 isoforms (a, b, c, and d), **Figure 1C**. They also share surface expression of many NK activating and inhibitory receptors, such as activating and inhibitory KIR, NKG2A-D, TIGIT, and PD-1. As such, distinct from αβT cells, iNKT cells can become activated in a TCR-independent fashion by way of their NK-like receptors, or in a TCR-dependent fashion when encountering target cells expressing CD1-lipid complexes. While comprised of a much more limited repertoire of TCR α and β chains compared to conventional αβT cells, there is still some variability in iNKT TCR chain usage. Still a debated topic, some iNKT subsets have been defined based on the specific TCR they express (128). Perhaps the best studied iNKT subset is restricted to the recognition of CD1d presenting glycolipids and uses a more invariant TCR comprised of a Vα24 (TRAV10)-Jα18 (TRAJ18) alpha chain in humans paired with a few possible Vβ chains (most commonly Vβ11). These iNKT cells are highly reactive to the marine-sponge derived glycolipid α-GalCer when presented by CD1d. Type II iNKT cells express a more heterogeneous, yet still CD1d-restricted, TCR which responds to various CD1d-presented glycolipids but not α-GalCer, and appears to be more immunoregulatory than Type I iNKT cells (129). Additional subsets have been defined in mice based on transcription factor usage, such as an iNKT17 subset that expresses RORγt, and secretes large amounts of IL-17 when stimulated (130). In the same study, iNKT1 cells produced more IFNγ, while iNKT2 cells produced more IL-4. Whether the same and/or other subset phenotypes exist in humans, and whether iNKT types are restricted lineages or can switch phenotypes is an active area of research. In humans, iNKT cells can be phenotypically divided into CD4, CD8, double-negative and double-positive subsets that respond differently to α-GalCer stimulation (131). CD4 single-positive iNKT cells are the most common, representing about 50% of iNKT cells in circulation, and are thought to be most similar to the iNKT2 population in mice, while CD8 single-positive iNKT cells are more similar to the iNKT1 population in mice (132). Under steady-state conditions, iNKT cells represent a very small fraction of circulating lymphocytes, typically less than 1% of the CD3+ population, and are found in various tissues including the bone marrow, spleen, and liver, as well as the lungs (133). In these tissues they are thought to play roles in surveillance against infections, tissue damage, and cancer. iNKT cells exhibit a response to stimuli that is kinetically distinct from NK cells and conventional T cells (134). This has been studied *in vitro* and *in vivo* in mice in response to α-GalCer as well as other Type I and Type II iNKT stimuli including bacterial pathogens, toll-like receptor TLR agonists, and sufatide. After stimulation, iNKT cells down-regulate their TCR and proliferate up to 10-fold, then contract through Bcl-2-mediated apoptosis and become anergic for a period of up to 2 months. Very limited reports of KLRG1+ long-lived effector iNKT cells in the lung have been described in response to α-GalCer stimulation, but an enrichment for specific CDR3 sequences was found suggesting a true although limited clonal memory population similar to that created after αβTCR engagement (135). Studies examining if cancer-specific stimuli result in similar iNKT response kinetics and memory have not been performed.

### iNKT cells in AML patients and intrinsic anti-AML responses

Many studies have described the innate ability of iNKT cells to respond to a wide variety of cancer cells in a wide variety of contexts, reviewed extensively elsewhere (136). However, only a few studies have described iNKT phenotypes and functions in AML patients. A crucial step in any anti-leukemic response is the presence of a leukemia-specific immune-activating target. While a variety of glycosphingolipids have been implicated as natural targets for iNKT stimulation in solid tumors (137), only one correlative study found a class of lipids called peroxisome-derived lipids were increased in the bone marrows of leukemia patients compared to healthy controls (138), and another showed increased lactotriaosylceramide, a glycosphingolipid, in bone marrow of AML patients particularly of the M1 phenotype (without maturation) (139). CD1d is consistently expressed on myelomonocytic (M4 and M5) leukemias and variable on other subtypes (140). Enrichment of specific glycolipids has not been demonstrated in bone marrow of M4 or M5 leukemia patients. This data is suggestive of but does not fully delineate the mechanism by which iNKT cells could recognize leukemic cells. Regarding activity, Boeck et al. found that leukemic patients had decreased iNKT signatures in PBMCs compared to healthy controls, but that a higher or preserved iNKT presence correlated with better outcomes in AML patients (141). Lower CD1d levels on monocytes was observed in peripheral blood of AML patients compared to healthy controls, and increased cytotoxicity of iNKT cells correlated with CD1d levels (142). Together this suggests an iNKT selective pressure against CD1d-high expressing myeloid cells in leukemic patients, but remains a correlative association. With advances in iNKT identification, further studies are needed to better understand the relationship between iNKT cells and leukemogenesis.

### iNKT cell use in therapies for AML

iNKT-based therapies to help treat AML are in the early nascent stages of development. Many, especially CAR-iNKT cells and the engineering of iNKT TCRs into T cells, have been studied for other malignancies with promising early results (143–146). In these approaches, the synthetic receptor provides deliberate antigen-targeted activation, but is complemented by cancer CD1d-targeting of the endogenous TCR of the iNKT cells. This dual activation is thought to be a beneficial and synergistic aspect specific to iNKT cells, potentially superior to the random array of endogenous TCRs expressed in αβTCR cell products. The limited frequency of iNKT cells has presented a practical challenge to generating large enough quantities for therapeutic applications. However, several groups have now defined efficient purification and expansion protocols for iNKT cells. In mice, splenocytes are subjected to CD5+ magnetic-bead purification, then iNKT cells are sorted by flow cytometry based on α-GalCer staining and cultured with plate-bound TCR stimulation and murine cytokines including IL-7 (147). This approach results in 10 iNKT cells in under 2 weeks of culture. In humans, various iNKT purification protocols exist, but typically involve α-GalCer selection or stimulation with α-GalCer loaded, irradiate PBMCs serving as APCs, and some combination of cytokines including IL-2 and IL-15 (148, 149). Li et al. developed a similar protocol to derive and expand highly pure iNKT cells from CD34+ precursors and showed they could be engineered to express a CAR, and then frozen for use in various patients (8, 150).

Ex vivo expanded iNKT cells have been utilized in different human leukemia-specific applications. Stavrou et al. showed that ex vivo expanded human iNKT cells recognized and killed AML cells in a CD1d-TCR-dependent manner, and also helped restore anti-leukemic activity of T cells despite an immunosuppressive leukemic microenvironment (151). Other studies examined anti-leukemic activity, as well as effects of iNKT cells on GHVD simultaneously. Schmid et al. demonstrated that ex vivo expanded and activated iNKT cells could directly lyse patient-derived leukemia cells, as well as decrease the activation and proliferation of alloreactive T lymphocytes (152). Third-party HSC-derived iNKT cells were shown to ameliorate GVHD in mouse xenograft models of human leukemia by eradicating APCs, but did not impair the GVL effect (153). This pre-clinical data is supported by clinical data showing high levels of iNKT cells in donor grafts correlated with lower GVHD risk but no increase in relapse (154, 155). In a Phase II study of 29 patients, Chen et al. injected a clinical grade α-GalCer formulation pre-SCT to attempt to stimulate iNKT cells in the grafts. They did not observe changes in iNKT numbers, but did see an increase in donor Tregs, suggesting iNKT cells may reduce GVHD indirectly by boosting tolerogenic Treg levels (156). Thus not only do iNKT cells possess a unique CD1d-specific TCR with inherent anti-leukemic activity, they may also help suppress GVHD. Although preliminary, these studies support further exploration of iNKT applications for both anti-GVHD and increased GVL effects.

## Gamma delta T cells in AML

### γδT cell biology

γδT cells are an innate-like subset of T cells expressing a TCR comprised of gamma and delta subunits, instead of alpha and beta subunits (**Figure 1D**). Like alpha and beta TCRs, the gamma and delta subunits are encoded by diverse V, D, and J gene repertoires that undergo somatic rearrangement to form unique TCR compositions. γδT cells express a combination of Vγ TCR chain classes (Vγ2, 3, 4, 5, 8, 9, and 11) and Vδ chain classes (Vδ1, 2, 3, and 5) (157). γδT cells are the first and dominant lymphocyte generated during fetal development, emerging in the liver even before the thymus is formed (158). In adults, γδT cells comprise less than 10% of circulating T cells, but remain enriched in mucosal tissues such as skin, gastrointestinal tract, genitourinary tract, and lung. Most γδT cells are CD4−CD8−, while approximately 30% express CD8 and <1% express CD4. Due to their genetic composition, γδTCRs are theoretically more polymorphic than αβ TCRs, with more rearrangement combinations of V, D and J subunits. However, our current knowledge supports that γδT cells recognize a limited subset of antigens, many of which are derived from stressed or malignant self-cells. The limited antigen recognition of γδT cells can be attributed to the TCR progression to oligoclonality over time, despite a highly diverse TCR repertoire originally present in the fetus. It is believed that both genetic and microenvironmental factors shape the emergence of dominant clones, although these mechanisms remain poorly understood. Like iNKT cells, γδ T cells are characterized as a hybrid component of the innate and adaptive immune systems due to their unique phenotypes and diverse functions. γδT cells recognize antigen targets in a mechanism unique to the classical topological αβ TCR-MHC interactions. In fact, antigen recognition by γδT cells is not inherently MHC-dependent. γδT cell clones with unique variable regions have a diverse array of antigen specificity, ranging from MHC class-I-like proteins to bacterial-associated heat shock proteins or tetanus toxin antigens. The best studied ligands for human Vγ9Vδ2 TCR are phosphoantigens, which are recognized in a BTN2A1-/BTN3A1-dependent manner. Interestingly, the mode of antigen recognition is unlike αβ or iNKTs T cells. The TCR on αβ or iNKT cells recognize antigens presented directly by MHC or CD1d molecules expressed on the cells’ surface. In sharp contrast, intracellular phosphoantigens bind to cytoplasmic tails of BTN2A1 or BTN3A1 molecules which induce structural changes to BTN2 and BTN3 on the cells surface. This conformational changes to extracellular BTN2A1 or BTN3A1 molecules are then recognized by the Vγ9Vδ2 TCR. Additional ligands for the Vγ9Vδ2 TCR include the DNA repair protein human MutS homologue 2 (hMSH2) and F1-ATPase when expressed together with apolipoprotein A-I.

The γδT cell response to bacterial infection occurs in distinct temporal stages, characterized as early or late responses, which have unique functional profiles. γδT cells in the early response arise from the liver and stimulate an inflammatory response by producing pro-inflammatory cytokines such as IFN-γ. Preclinical cancer studies have identified γδT cells as one of the earliest sources of IFN-γ that, in turn, can then promote MHC I expression on αβT cells and their subsequent anti-tumor activity (159, 160). γδT cells in the late response to bacterial infection arise from the lymphoid system and suppress the inflammatory response by producing anti-inflammatory cytokines, such as IL-10. These data support that γδT cells have diverse and sometimes conflicting responses that are context and time dependent, which should be considered in the development of anti-cancer therapeutic approaches.

Preclinical studies have revealed that mice lacking γδT cells are highly susceptible to multiple carcinomas (161), supporting the anti-tumor activity of γδ T cells. Importantly, murine studies have also show evidence of γδT cell memory responses following infection (162, 163), which may have important implications for durable anti-tumor clinical responses and reduced recurrences. Additionally, it notable that not all γδT cells have the same anti-tumor effects. For example, IL-17-producing Vγ5δT cells have decreased perforin expression and exhibit pro-tumor effects in mouse models (164). Therefore, the composition of γδT cells used in therapeutic approaches for patients requires careful consideration, and better characterization of γδT cells in AML is warranted.

### γδT cell status in AML patients

Similar to αβT cells, activated γδT cells can elicit anti-tumor cytotoxic activity in AML (165). Direct mechanisms include secretion of granzyme B and perforin, induction of apoptosis in target cells, and cytokine (e.g., IFN-γ and TNF-α) production, while indirect mechanisms include cross talk with other immune cells, such as B cells and dendritic cells. Recent studies have reported that γδT cells are an abundant component of tumor infiltrating lymphocytes (TILs) in a multitude of cancer indications, including AML. Patient-derived γδT cells exhibit a large amount of TCR variability, both within individuals and across different cancer types. γδT cells do not correlate with intra-tumor αβT cells in patients (166). Increased γδT cells have been associated with higher disease-free and overall survival in leukemia patients following αβ-depleted allogeneic bone marrow transplantation (167, 168). More recently, specific γδT cell variants have demonstrated potent anti-tumor efficacy in AML. For example, patient-derived Vγ9Vδ2T cells can recognize and kill AML blasts ex vivo via release of perforin and granzyme B in a TCR-dependent manner. Notably, Vγ9Vδ2T cells from AML patients had a strong effector memory phenotype with high cytotoxic capacity and reduced proliferative capacity, whereas Vγ9Vδ2T cells from healthy volunteers had a strong central memory phenotype with high proliferative capacity and reduced cytotoxic capacity (169). These data suggest that AML cells can reprogram γδT cells and cause dysfunction by reducing their downstream expansive capabilities. It also suggests that γδ TCR chain classification may be an insufficient readout of anti-tumor function and functional markers could be required.

### γδ T cells as adoptive cell therapy for AML

The first studies of γδT cells in STC occurred in the 1990s (170, 171), (**Figure 2**) but the anti-tumor effects of γδT cells have yet to be fully exploited for AML patients. An initial challenge was overcoming the low apoptotic threshold of γδT cells, which can be accomplished with the exogenous IL-2 treatment that is now standard protocol (172). Current strategies are focused on utilization of Vδ1 or Vδ2 subsets, primarily in allogenic applications due to the inherent reduced risk of GVHD with HLA-independent γδT cells.

An ongoing challenge includes the difficulty of expanding γδ T cells *in vivo* or ex vivo feasibly on a large sale. Yazdanifar et al. present a comprehensive summary of *in vivo* and ex vivo approaches to optimize γδ Tcell expansion in multiple cancer types (173), and several recent studies have focused on viable approaches to expand γδT cells for AML applications. Ex vivo approaches involve isolating γδT cells from PBMCs and stimulating them with phosphoantigen (pAg) or bisphosphonates (usually zoledronic acid), while *in vivo* approaches involve stimulation with pAg or nitrogenous bisphosphonate. Xiao et al. reported that ex vivo stimulation with zoledronic acid, IL-2, and gamma-irradiated, CD64-, CD86- and CD137L-expressing K562 APCs results in significant donor-derived γδT cell expansion (174). Boucher et al. also reported that ex vivo culture with zoledronic acid, IL-2, and genetically engineered K-562 CD3scFv/CD137L/CD28scFv/IL15RA APCs markedly expands donor-derived γδT cells. This approach resulted in a 633-fold expansion of γδ T cells that exhibited a 43% effector memory phenotype, indicating the potential for a robust and long-lived anti-tumor response. Even after a freeze-thaw cycle, these cells were highly cytotoxic and able to eliminate Chinese hamster ovary cells in culture (175). Both of these studies were performed in a Good Manufacturing Practice (GMP) compliant manner, making these approaches more commercially feasible.

Another limitation of γδ T cells is the potential of conversion to an exhausted/anergic phenotype after persistent antigen stimulation, like αβT cells. The γδT cell exhaustion signature included increased expression of immune checkpoint proteins, decreased cytokine production, and decreased effector function. In AML patients following allogeneic stem cell transplantation, exhausted γδT cell exhibited high PD-1 and TIM-3, which corresponded with reduced TNF-α and IFN-γ expression (176). Inhibition of PD-1 and/or TIM-3 may block or revert exhaustion of γδT cells and warrants investigation as combination therapy in future studies. Another application of γδT cells as an adoptive therapy for AML includes CAR approaches. γδT cells were first successfully transduced with CAR genes in the early 2000s, and they demonstrated potent anti-tumor activity and produced high levels of IFN-γ (177). In addition to their anti-tumor activity, γδT cells transduced with a CD19-specific CAR are less prone to exhaustion (178), suggesting that the use of CAR γδT cells may reduce concerns about T cell exhaustion in patients and result in a more long-lived anti-tumor response.

There are currently several clinical trials are ongoing to investigate the potential of γδT cells as adoptive therapy in AML and other hematologic malignancies. While γδT cells have been investigated in autologous cellular applications, most trials utilize allogenic applications. One Phase 1 trial is utilizing a single infusion of allogeneic γδT cells, which are first expanded ex vivo with zoledronic acid and IL-2, followed by post-transplant cyclophosphamide (NCT03533816). A second Phase 1/1b trial, focused solely on AML, is utilizing a similar protocol consisting of a single infusion of allogeneic γδT cells after ex vivo stimulation and expansion with artificial antigen presenting cells (AAPCs) (NCT05015426). Both trials list goals of maximizing the anti-tumor immune response and simultaneously minimizing the risk of GVHD complications, and both trials are still recruiting with estimated primary completion dates in 2024. A Phase 2 trial in adolescents (<21) with hematologic malignancies, including AML, is also underway investigating the efficacy of αβTCR-depleted allogeneic cell transplantation (thus enriched for γδT cells) followed by a second infusion of CD45RA-depleted donor memory cells (NCT03849651). Because γδT cell therapies are reported to reduce the risk of GVHD compared to other T cell adoptive therapies (179, 180), these trials are expected to be well-tolerated and limit transplant-associated mortality.

There are also alternative therapeutic approaches in development to harness the anti-tumor ability of γδT cells. One such approach is the activating antibody LAVA-051, which is a bispecific antibody that engages Vγ9Vδ2 on T cells to target CD1d-expressing tumor cells. A phase 1/2a trial is currently in progress in patients with relapsed/refractory AML and other blood cancers (NCT04887259). Another ongoing approach is to transduce the γδTCR into αβT cells, then expend them with αβ-optimized protocols. One such candidate is “TEG001” which expresses the Vγ9Vδ2TCR, recognizes CD277 antigen on AML cells, and can eliminate AML blasts in preclinical models (181). A clinical trial investigating TEG001 in the Netherlands is currently ongoing (NTR6541). A final approach is the Delta One T (DOT) cell-generating protocol, which includes a three-week TCR stimulation with cytokines (anti-CD3 antibody with IL-4, IFNγ, IL-21, and IL1-β) and generates Vδ1T cells that express cytotoxicity-associated NK cell receptors (NKG2D, DNAM-1, NKp30, and NKp4) and can eliminate human AML xenografts (182). The DOT protocol is also being investigated with CAR γδT cells that target the AML antigen CD123 (183), and optimistically will be tested in clinical trials in the near future. Taken together, the increasingly optimized *in vivo* and ex vivo expansion protocols, as well as methods to minimize exhaustion such as using CAR approaches, support that γδT cells are a promising adoptive cell therapy approach for patients with AML that could have successful anti-GVHD and increased GVL applications.

## Conclusions

While most current adoptive cell therapies for AML rely on conventional αβT cells, mounting evidence supports the use of NK, iNKT, and γδT cells in specific applications. Despite being less abundant in steady state conditions, NK, iNKT, and γδT cell types can now be readily expanded and engineered ex vivo to levels required in adoptive therapies. Distinct from αβT cells, NK, iNKT and γδT cells have potential for universal applications due to lack of HLA-restriction. KIR-HLA-mismatching of NK cells can be exploited to target and destroy residual host leukemic cells. Regarding iNKT cells, their semi-variant CD1d-specific TCR can theoretically be functional in any recipient and provides inherent anti-leukemic activity. While maintaining the ability to recognize conventional peptide-MHC, the γδTCR can also recognize conserved antigens and molecules associated with stressed self. Remarkably, all three appear to have anti-GVHD properties, in sharp contrast to the GVHD-inducing effects of αβT cells. Preliminary adoptive NK, iNKT, and γδT cell approaches exhibit limited expansion and short lifespans *in vivo*, however new ex vivo conditioning and engineering approaches are under development to address these issues. Finally, their relative plasticity, under unfavorable conditions, could result in phenotypes detrimental to the host. Further understanding and careful application of NK, iNKT, and γδT cells could be extremely beneficial for AML patients in the near future.

## Author contributions

AK wrote the manuscript and prepared the figures and tables. LC co-wrote and reviewed the manuscript. ED oversaw the entire project providing guidance as well as detailed review and editing of all aspects of the paper. All authors contributed to the article and approved the submitted version.
